# Case Report: A rare case of gallbladder carcinosarcoma with osteosarcomatous differentiation

**DOI:** 10.3389/fonc.2025.1692825

**Published:** 2025-11-20

**Authors:** Jiajun Shi, Miao Shen, Jie Yao, Xiangxiang Shen, Zhengrong Su

**Affiliations:** Deqing People’s Hospital (Sir Run Run Shaw Hospital, School of Medicine, Zhejiang University), Zhejiang, Huzhou, China

**Keywords:** gallbladder carcinosarcoma, osteosarcomatous differentiation, AFP, differential diagnosis, epithelial, mesenchymal

## Abstract

Gallbladder carcinosarcoma (GBCS) is indeed an exceptionally rare and aggressive malignancy, accounting for less than 1% of all primary gallbladder tumors. This tumor is characterized by the presence of both carcinomatous (epithelial) and sarcomatous (mesenchymal) components, making it a unique and diagnostically challenging entity. Herein, we report a case of a 71-year-old female patient who presented with a one-year history of vague epigastric pain. AFP levels were markedly elevated. Preoperative imaging revealed gallbladder enlargement with heterogeneous contrast enhancement, raising suspicion of malignancy. The patient subsequently underwent radical cholecystectomy, and postoperative histopathological examination confirmed the diagnosis of GBCS with osteosarcomatous differentiation. The patient has since completed her first cycle of chemotherapy. Even with radical resection and adjuvant chemotherapy, GBCS carries a grave prognosis. Heightened clinical suspicion, thorough imaging assessment, and confirmatory histopathological biopsy are essential for accurate preoperative diagnosis. Surgical optimization and personalized treatment strategies remain critical to improving outcomes.

## 1 Introduction

Gallbladder carcinosarcoma (GBCS) is an exceptionally rare and aggressive malignancy of the digestive tract, representing fewer than 1% of primary gallbladder tumors (1). Histologically, it exhibits biphasic differentiation, combining epithelial and mesenchymal elements, with adenocarcinoma as the most frequent epithelial component and spindle cells predominating in the mesenchymal portion; osteosarcomatous differentiation is particularly uncommon (2). Multidisciplinary approaches, including neoadjuvant therapies and targeted treatments, are being investigated to improve outcomes. Increased awareness and reporting of cases are essential to advance research and develop effective therapeutic protocols (3, 4).

This report details the clinical manifestations, radiological characteristics, and pathological features of GBCS. As current evidence consists largely of sporadic case reports and no formal diagnostic or therapeutic guidelines exist, this study aims to enhance the understanding of this rare malignant tumor and to offer a reference for clinical management in comparable cases.

This case report documents a 71-year-old female patient presenting with abdominal pain who was diagnosed with GBCS after radical cholecystectomy. The tumor displayed mesenchymal differentiation into osteosarcomatous components and was associated with markedly elevated AFP levels, which is exceptionally rare among cases and further underscores the uniqueness of this case (5). The confluence of this rare entity and its unusual clinicopathological characteristics renders this case highly instructive for improving diagnostic and therapeutic approaches to GBCS.

## 2 Case report

A 71-year-old female farmer with no prior medical history was admitted on June 23, 2025, reporting persistent dull epigastric pain for one year, occasionally accompanied by nausea and vomiting. Physical examination showed abdominal symmetry without distension, with mild epigastric tenderness but no Murphy’s sign.No scleral icterus or cutaneous jaundice was observed.

Laboratory tests indicated hepatic dysfunction, with ALT (205 IU/L), AST (127 IU/L), ALP (374 IU/L), GGT (369 IU/L), and LDH (341 IU/L) all elevated, while bilirubin remained normal. Notably, AFP levels reached 2,786 μg/L.

**Figure 1:**
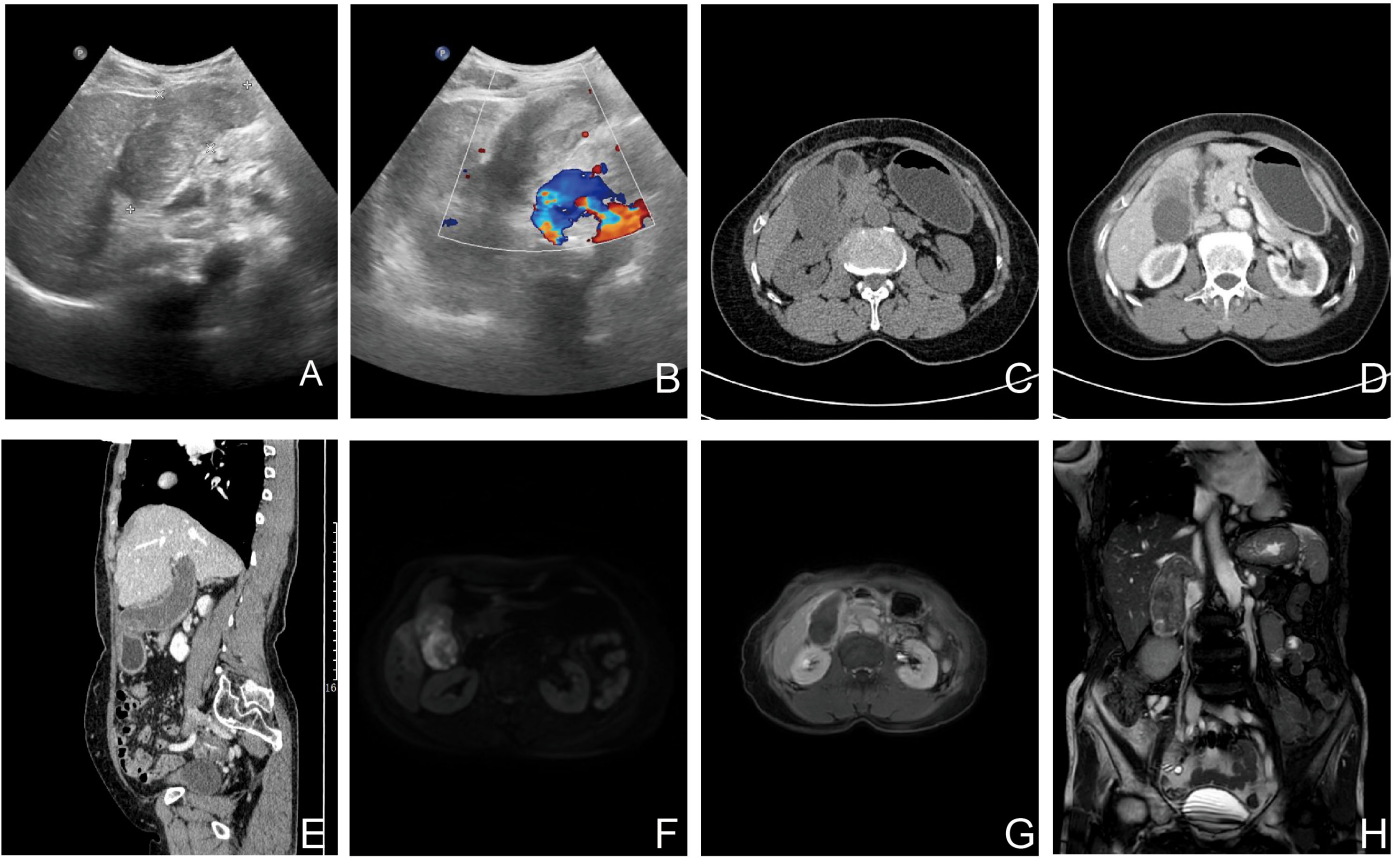
Preoperative imaging of the GBCS (Ultrasound, CT and MRI). Color doppler ultrasound images: **(A, B)** The images show a hypoechoic mass with well-defined margins. The Doppler signals (red and blue) demonstrate the vascular distribution around the lesion. These findings have some value in the preoperative differentiation between benign and malignant lesions but are non-specific. **(C-E)** Unenhanced and contrast-enhanced CT demonstrate an enlarged gallbladder with diffuse wall thickening and enhancement upon contrast administration. **(F-H)** Diffusion-weighted imaging (DWI), contrast-enhanced T1-weighted, and T2-weighted coronal images show gallbladder wall thickening accompanied by enhancement and restricted diffusion. These findings are highly suggestive of malignancy.

Ultrasonography detected an 8.3 × 3.6 × 4.5 cm hypoechoic mass with well-defined margins in the gallbladder fossa. Contrast-enhanced abdominal CT revealed gallbladder enlargement with irregular wall enhancement, suggesting adjacent hepatic inflammation, along with suspicious soft tissue thickening in the common bile duct and gallbladder neck. Abdominal MRI further confirmed irregular gallbladder wall enhancement, nodular enhancement at the neck (suspicious for malignancy), and abnormal hepatic perfusion (**Figure 1**).

The patient’s medical history and diagnostic evaluations led the Hepatobiliary Surgery team to suspect a gallbladder tumor preoperatively. On June 27, 2025, surgeons performed laparoscopic cholecystectomy, revealing marked gallbladder wall edema (12×6×4 cm) and a 4×4 cm fundal mass. The resected specimen underwent pathological assessment, with frozen section analysis confirming malignancy. After consulting the patient’s family, the surgical team proceeded with open radical cholecystectomy to address the gallbladder cancer.

**Figure 2:**
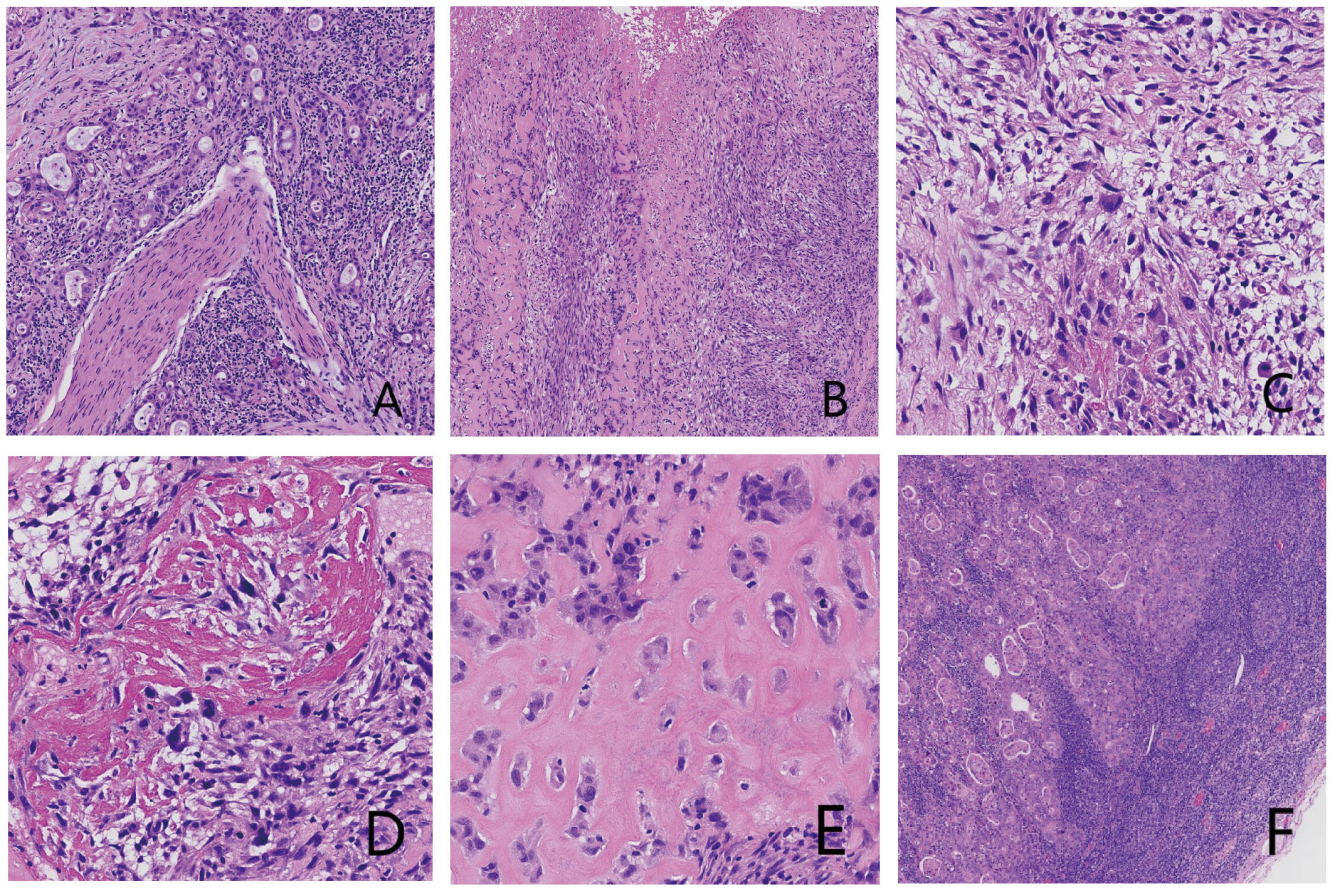
Histopathological micrographs of tumor tissue with hematoxylin and eosin (H&E) staining: **(A)** Adenocarcinoma component. Central adenocarcinoma tissue with perineural invasion is observed. **(B)** Mesenchymal component, showing cellular dense and loose areas. **(C)** Lower magnification of the mesenchymal component. Spindle-shaped cells with eosinophilic and pale cytoplasm are present; a multinucleated giant cell is visible in the center. **(D)** Ossified area. **(E)** Eosinophilic osteoid matrix with polygonal cells exhibiting hyperchromatic nuclei. **(F)** Lymph node metastasis composed of adenocarcinoma.

**Figure 3:**
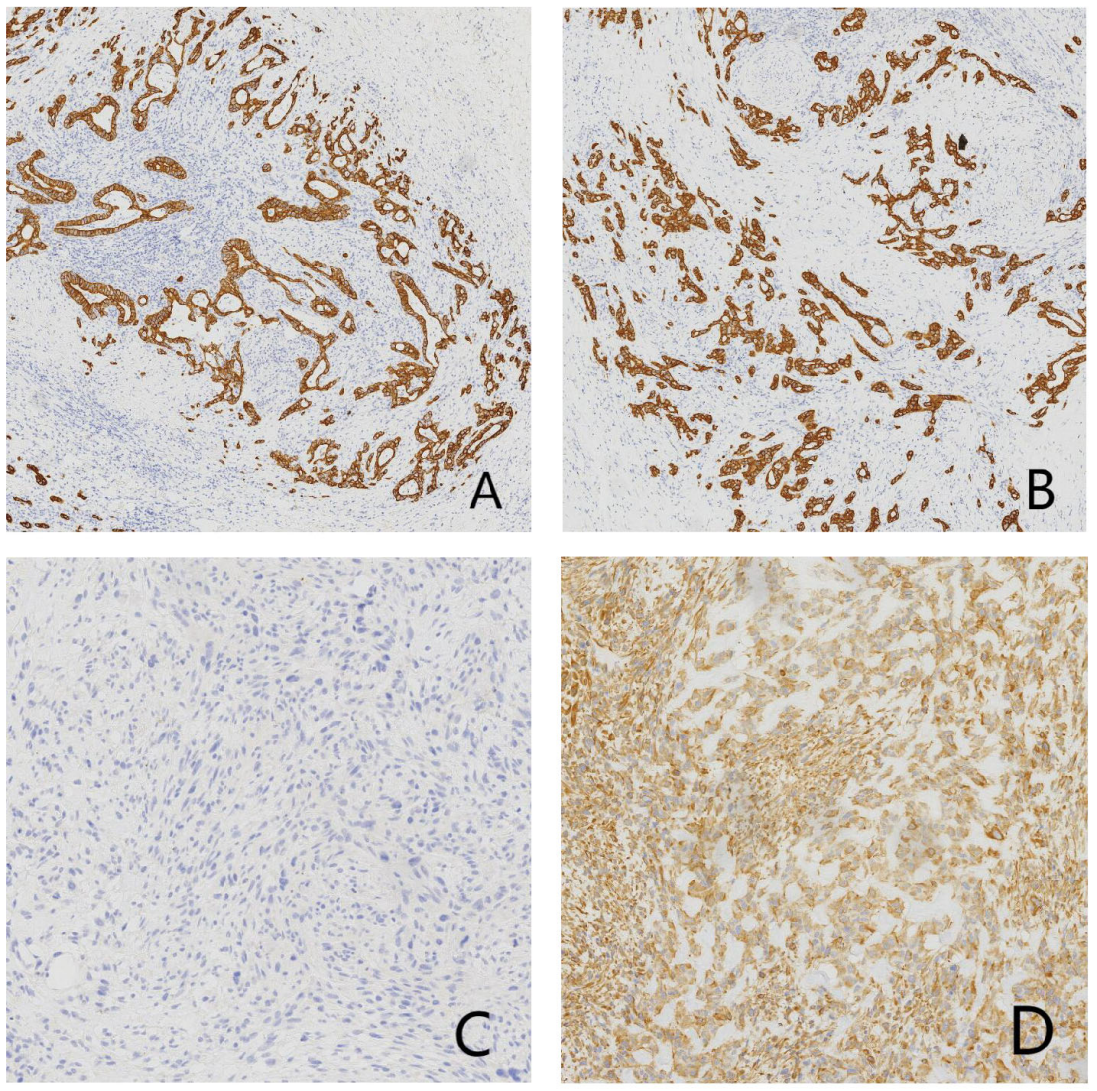
Immunohistochemical (IHC) micrographs of tumor tissue: **(A)** Positive expression of CK in the carcinomatous component. **(B)** Positive expression of CK19 in the adenocarcinoma component. **(C)** Negative expression of CK in the mesenchymal component. **(D)** Positive expression of vimentin in the mesenchymal component.

Gross examination showed a gallbladder measuring 12.0 cm × 5.5 cm × 4.5 cm with diffusely thickened, indurated walls (approximately 1.5 cm). The cut surface appeared gray-white with a roughened mucosa, while the serosal surface adhered to hepatic tissue (5 cm × 4.5 cm × 3.0 cm), displaying a gray-yellow cut surface interspersed with focal gray-white areas. Histopathological examination revealed a tumor composed of epithelial carcinoma and mesenchymal elements. The carcinoma component exhibited atypical glandular structures, cords, and nests with marked cellular pleomorphism, nuclear atypia, and frequent pathological mitoses, consistent with adenocarcinoma by immunohistochemistry. The mesenchymal component alternated between densely packed blue-stained cellular zones and loosely arranged pink-stained regions. The dense areas contained spindle cells arranged in fascicles or sheets, whereas the loose areas featured round to polygonal cells embedded in abundant pink osteoid-like matrix. Transitional zones between these components were present, with morphology and immunoprofile (vimentin-positive, CK-negative) supporting osteosarcoma. Histopathological examination (HPE) identified a diffusely infiltrating malignant gallbladder tumor exhibiting morphological and immunohistochemical characteristics of carcinosarcoma, with both poorly differentiated adenocarcinoma and osteosarcoma components. The tumor breached the serosal layer and extended into the adjacent hepatic parenchyma, with lymph node metastasis detected in 1 of 6 examined lymph nodes (Station 13: 1/1; Stations 8 and 12: 0/5) (**Figure 2**). Immunohistochemical (IHC) analysis yielded the following results: The adenocarcinoma component expressed CK, CK7, CK19, and CEA, with focal GPC3 positivity. The osteosarcoma component showed diffuse vimentin expression and focal S-100 reactivity. No immunoreactivity was observed for myogenin, CD117, or DOG1. CD34 and D2–40 selectively labeled vascular and lymphatic structures, respectively. The Ki67 proliferation index peaked at 80% in hotspot areas (**Figure 3**).

The patient’s postoperative symptoms improved significantly, with epigastric distension and pain substantially alleviated, and no vomiting, nausea, or palpitation observed. The patient was discharged on July 13, 2025. The first cycle of chemotherapy was completed on July 31, 2025, followed by discharge(specific regimen: gemcitabine 1000 mg D1, 8 + carboplatin 300 mg D1). During follow-up to date, the patient has maintained good general condition with no evidence of metastatic lesions. Further follow-up is required for ongoing monitoring.

## 3 Discussion

GBCS represents an exceptionally rare malignancy, with limited clinical experience and poorly characterized pathophysiology. This aggressive tumor predominantly occurs in middle-aged and elderly patients, showing a female predominance and frequent association with cholelithiasis (1, 6). Clinical presentation typically involves nonspecific right upper quadrant pain and abdominal masses, though some patients develop fever, jaundice, or gastrointestinal symptoms (7, 8). Advanced cases often demonstrate direct organ invasion or distant metastasis through lymphatic and hematogenous routes (3, 9). The current case involved a 71-year-old woman whose mild epigastric pain represented the sole clinical manifestation, without accompanying cholelithiasis or jaundice—factors that likely contributed to diagnostic delay.

GBCS lacks reliable serum biomarkers or specific radiological characteristics, complicating preoperative diagnosis (10). Most reported cases indicate that CEA levels typically remain normal or mildly elevated, while CA19–9 is often moderately elevated, generally below 100 IU/mL (11, 12). In contrast, our patient exhibited a normal CA19–9 level (28.2 IU/L) but a significantly elevated AFP level. A previous case reported an AFP-producing gallbladder carcinosarcoma with a level of 1,495 ng/mL, while the level in our case was even higher (2,786 μg/L), potentially associated with hepatic invasion by the gallbladder tumor. Follow-up revealed a rapid decline in serum AFP levels after resection, with the current level at 8.51 μg/L, approaching the normal range (<7 μg/L)(**Supplementary Figure S1**). Some scholars suggest that AFP-producing gallbladder carcinomas metastasize more frequently to the liver and have a poorer prognosis compared to those not producing AFP (13).

Radiologically, the gallbladder often exhibits mild-to-moderate heterogeneous persistent enhancement, most commonly localized at the fundus of the gallbladder, followed by diffuse involvement of the entire organ (14). Due to the paucity of early clinical symptoms and nonspecific radiological characteristics, early diagnosis of gallbladder carcinosarcoma is challenging, and definitive diagnosis largely relies on postoperative pathological examination. Initial laparoscopic cholecystectomy plans were revised to radical resection after intraoperative frozen section revealed malignancy, underscoring pathology’s decisive role in surgical management. Histopathological examination identified an osteosarcomatous component within the sarcomatous tissue, representing the rarest subtype.

In pathological diagnosis, GBCS must be differentiated from benign lesions and sarcomatoid carcinoma. The distinction from benign lesions is relatively straightforward based on the presence of cellular atypia. Sarcomatoid carcinoma exhibits two morphological patterns: spindle cell type and giant cell type. In the present case, the densely cellular areas demonstrated spindle-shaped morphology with frequent giant cells, necessitating differential diagnosis. GBCS is characterized by two distinct components—carcinoma and sarcoma—without intermingling or poorly defined tissue boundaries. In contrast, sarcomatoid carcinoma shows indistinct demarcation between carcinomatous and sarcomatoid components (15, 16). Immunohistochemical markers for epithelial and mesenchymal differentiation further aid in differentiation. Sarcomatoid carcinoma, being epithelial in origin, expresses epithelial markers such as cytokeratin (CK) in all components. Conversely, carcinosarcoma demonstrates both epithelial and mesenchymal differentiation, with the mesenchymal component expressing markers like vimentin while lacking epithelial marker expression (17–19). Additionally, the prominent glandular differentiation and positive CK7/CK19 expression in this case excluded the diagnosis of undifferentiated carcinoma. Microscopically, the marked cellular atypia facilitated differentiation from gallbladder adenomyomatosis. Moreover, the clear demarcation between carcinomatous and sarcomatous components, absence of transitional zones suggestive of metastasis, and corroborative immunohistochemical findings confirmed the definitive diagnosis of GBCS.

With a median survival of 5.5 months, GBCS carries a dismal prognosis, though radical resection improves 5-year survival to 31%, particularly for early-stage disease (7, 20). While adjuvant chemotherapy is routinely recommended, no standardized regimen exists, and treatment efficacy remains unproven. Targeted treatments are being investigated to improve outcomes (21, 22).The patient received gemcitabine-carboplatin therapy postoperatively (23), tolerating the first cycle well, with ongoing follow-up.

## 4 Conclusion

GBCS with osteosarcomatous differentiation is an exceptionally rare malignancy whose pathophysiology remains poorly understood. The nonspecific clinical, laboratory, and imaging features frequently result in misdiagnosis before surgery. While optimal treatment strategies remain undefined, early-stage cases demonstrate the most favorable outcomes following surgical resection. Further case documentation would advance our knowledge of cancer epidemiology to improve diagnosis, therapeutics, and management strategies.

## Data Availability

The original contributions presented in the study are included in the article/Supplementary Material. Further inquiries can be directed to the corresponding authors.
