# Supplementary material for: Case Report: A rare case of gallbladder carcinosarcoma with osteosarcomatous differentiation
Source: Front Oncol. 2025 Nov 20;15:1692825. doi: 10.3389/fonc.2025.1692825 (PMC12675190; doi:10.3389/fonc.2025.1692825)
Supplement: Supplementary file 2 [file DataSheet2.docx]

Supplementary Material

Supplementary Figures and Tables

## Supplementary Figures

**Figure S1.** Graph of changes in tumor markers.


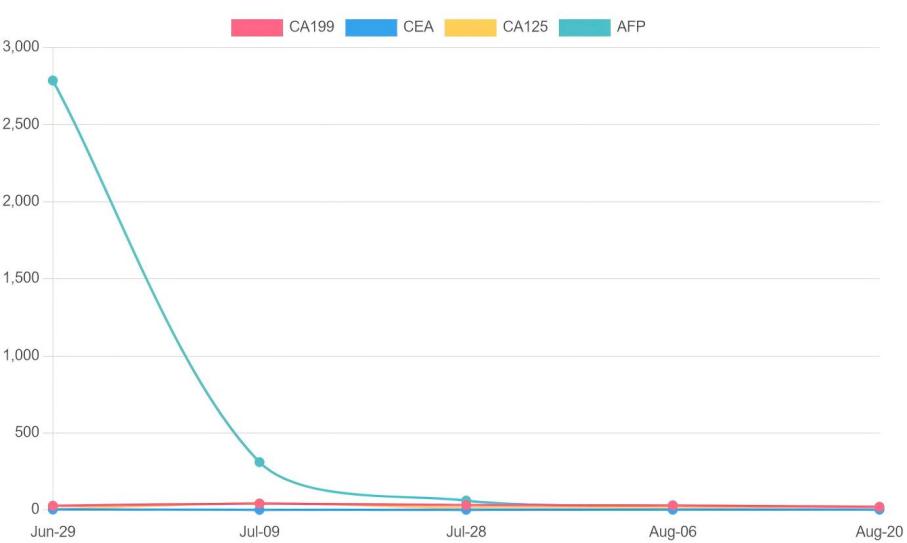


Table showing changes in specific tumor marker values. Red indicates abnormal values.

| Time | CA199 | CEA | CA125 | AFP |
| --- | --- | --- | --- | --- |
| ref | ＜30U/ml | ＜5ug/L | ＜24U/ml | ＜7ug/L |
| Jun-29 | 28.2 | 3.42 | 7 | 2786 |
| Jul-09 | 41.8 | 1.52 | 44.1 | 311 |
| Jul-28 | 32.3 | 1.89 | 14.2 | 61.72 |
| Aug-06 | 30.8 | 2.15 | 16.1 | 25.91 |
| Aug-20 | 21.4 | 2.36 | 10.6 | 8.51 |
